# Effect of Cyclic Adenosine Monophosphate on Connexin 37 Expression in Sheep Cumulus-Oocyte Complexes

**DOI:** 10.3390/jdb12020010

**Published:** 2024-03-27

**Authors:** Mengyao Zhao, Gerile Subudeng, Yufen Zhao, Shaoyu Hao, Haijun Li

**Affiliations:** 1 College of Veterinary Medicine, Inner Mongolia Agricultural University, Hohhot 010018, China; zhaomengyao@emails.imau.edu.cn (M.Z.); subudaimau@163.com (G.S.); 18686115060@emails.imau.edu.cn (Y.Z.); haoshaoyu@emails.imau.edu.cn (S.H.); 2 Inner Mongolia Key Laboratory of Basic Veterinary Science, Inner Mongolia Agricultural University, Hohhot 010018, China; 3 Key Laboratory of Animal Embryo and Development Engineering of Autonomous Region Universities, Hohhot 010018, China

**Keywords:** sheep COC, cAMP, Cx37, gene transcription, protein expression

## Abstract

Gap junctional connection (GJC) in the cumulus–oocyte complex (COC) provides necessary support for message communication and nutrient transmission required for mammalian oocyte maturation. Cyclic adenosine monophosphate (cAMP) is not only a prerequisite for regulating oocyte meiosis, but also the key intercellular factor for affecting GJC function in COCs. However, there are no reports on whether cAMP regulates connexin 37 (Cx37) expression, one of the main connexin proteins, in sheep COCs. In this study, the expression of Cx37 protein and gene in immature sheep COC was detected using immunohistochemistry and PCR. Subsequently, the effect of cAMP on Cx37 expression in sheep COCs cultured in a gonadotropin-free culture system for 10 min or 60 min was evaluated using competitive ELISA, real-time fluorescent quantitative PCR (RT-qPCR), and Western blot. The results showed that the Cx37 protein was present in sheep oocytes and cumulus cells; the same results were found with respect to GJA4 gene expression. In the gonadotropin-free culture system, compared to the control, significantly higher levels of cAMP as well as Cx37 gene and protein expression were found in sheep COCs following treatment in vitro with Forskolin and IBMX (100 μM and 500 μM)) for 10 min (*p* < 0.05). Compared to the controls (at 10 or 60 min), cAMP levels in sheep COCs were significantly elevated as a result of Forskolin and IBMX treatment (*p* < 0.05). Following culturing in vitro for 10 min or 60 min, Forskolin and IBMX treatment can significantly promote Cx37 expression in sheep COCs (*p* < 0.05), a phenomenon which can be counteracted when the culture media is supplemented with RP-cAMP, a cAMP-specific competitive inhibitor operating through suppression of the protein kinase A (PKA). In summary, this study reports the preliminary regulatory mechanism of cAMP involved in Cx37 expression for the first time, and provides a novel explanation for the interaction between cAMP and GJC communication during sheep COC culturing in vitro.

## 1. Introduction

Oocyte maturation represents a pivotal stage in the female reproductive cell development, characterized by intricate and nuanced processes. Throughout oocyte maturation, both the nucleus and cytoplasm undergo a series of alterations and regulations to ensure its proper development and fertilization competency [1]. The intercellular communication between cumulus cells (CCs) and the oocyte is essential for oocyte growth, allowing the transfer of nutrients and other small molecules between them and ensuring that the oocyte undergoes full development until fertilization [2,3].

Cyclic adenosine monophosphate (cAMP) is currently known as a core factor that regulates the nuclear maturation process of oocytes. Recent studies have revealed that cAMP can not only regulate oocyte nuclear maturation, but also plays an important role in its cytoplasm maturation and subsequent embryonic development. It is widely recognized that high levels of cAMP in cumulus–oocyte complexes (COCs), both in vivo and in vitro, are a prerequisite for maintaining oocyte meiosis arrest. cAMP is produced by adenylate cyclase (AC) after stimulating G-protein-coupled receptors, mainly through cAMP-dependent serine/threonine protein kinase A (PKA). PKA is considered to be the most important effector of cAMP. It consists of two catalytic subunits and two regulatory subunits. The latter combines with cAMP to form an active catalytic subunit to regulate protein expression [4]. In addition, the degradation of cAMP is achieved by phosphodiesterase (PDE). PDE regulates the level and signal of cAMP by hydrolyzing cAMP, thereby affecting cAMP-dependent processes [5]. In this way, AC and PDE cooperate with each other and jointly participate in the regulation of intracellular cAMP concentration, thereby maintaining a high level of cAMP or a low level of cAMP to restore the meiotic arrest of oocytes. Studies have shown that simply increasing the level of cAMP in multi-species oocytes during in vitro maturation can significantly promote the developmental ability of subsequent embryos, but the underlying mechanism is still not fully understood [6,7]. 

The gap junction communication among cells is very important for the information exchange and material exchange required for oocyte maturation. Connexin 37 (Cx37) and connexin 43 are the two most widely expressed and most important gap junction proteins in mammalian ovaries. These proteins form the gap junction channel among cells in the follicle in a homologous or heterologous manner, transporting various information materials needed to regulate oocyte growth and follicle development between oocytes and CCs [8]. Among them, it is known that Cx37 is a gap junction protein mainly expressed by oocytes. Although the expression of Cx37 in cumulus cells varies with species, the conclusion that Cx37 in oocytes is the direct cause of GJC changes between cumulus and oocytes has been confirmed by previous research [9]. Whether Cx37 antibodies are injected into the intact follicles or the Cx37 gene is knocked out in a mouse [10], the gap junctions on the surface of the oocyte will be eliminated [11].

Studies have reported that gap junction channels have an accumulation effect on the level of cAMP in oocytes. Interestingly, cAMP also has a regulatory function with respect to the gap junction function between cumulus and oocytes. Li Haijun et al. [12] found that adding Forskolin and IBMX can significantly prolong the open time of bovine COC channels and enhance the gap junction function. In another human study, increasing cAMP levels (Cilostamide and Forskolin) not only alleviated the loss of gap junctional connection (GJC), but also delayed the onset of GVBD, and enhanced the ability of oocyte development [13]. But how does cAMP conduct its regulatory effect? Whether this regulation is through the alteration of the expression or function of related gap junction proteins, especially Cx37, has not been established yet.

Therefore, in this study, immunohistochemistry, PCR, ELISA, qPCR, and Western blot techniques were adopted to explore the possible regulatory mechanism of cAMP in sheep COC with respect to Cx37 expression in a gonadotropin-free culture system. This was achieved by combining cAMP promotion reagents (Forskolin and IBMX) in order to provide experimental support to the determination of ovine oocyte maturation mechanisms and accelerate the utilization of oocyte matured in vitro in livestock production.

## 2. Materials and Methods

### 2.1. Sheep Cumulus-Oocyte Complex (COC) Collection

Ovaries were obtained from a commercial abattoir and transported to the laboratory in saline containing penicillin (10 U/mL) and streptomycin (10 µg/mL) at a temperature of 35–37 °C within 2 h. Immature COCs from antral follicles measuring 2–6 mm in size were obtained by slicing the ovaries and were then placed in tissue culture medium (TCM–199, Gibco, New York, NY, USA, 31100–027) supplemented with heparin sodium (Sigma, St. Louis, MO, USA, PHR8927, 0.1 mg/mL), gentamicin (Sigma, E003632, 0.05 mg/mL), sodium bicarbonate (Sigma, S5761, 2.2 g/mL), and bovine serum albumin (Solarbio, Beijing, China, BSA; A8010, 1 mg/mL) until they were transferred to an IVM or pre-IVM medium. Only COCs surrounded by multiple layers of compact CCs were used for the experiments. All animals were nonpregnant and studies carried out in vitro were subjected to the same culture conditions and treatments. All animals were cared for and handled following specific protocols approved by the Animal Welfare and Research Ethics Committee of the Inner Mongolia Agricultural University, China (approval no: NND2022001).

### 2.2. Indirect Immunofluorescence Staining

Cx37 location in sheep COCs was detected using the new COCs paraffin section technology established in our laboratory [11]. The specific steps were as follows: the group of COCs (40–50) was fixed as a unit in 4% paraformaldehyde (pH 7.4, PFA, 158127, Sigma-Aldrich) for 12 h. After fixation, the COCs were dehydrated by successive 30 min exposures to 70%, 95%, and 100% ethanol, incubated in xylene for 30 min, and embedded in paraffin. Sections (5 μm) were cut with a MICROM microtome Type Cool Cut (MICROM International GmbH, Walldorf, Germany) and collected on poly-L-lysine-coated slides (Sigma, USA). Paraffin sections were dewaxed by two 5 min exposures to xylene and absolute ethanol, followed by three washes for 5 min each with PBS (pH 7.4, P5368, Sigma-Aldrich).

Antibody incubation and stain processing: for sections, non-specific antibody binding was blocked in 5% normal donkey serum (NDS, O17-000-121, Jackson Laboratories, Bar Harbor, ME, USA) at room temperature for 1 h. After incubation with Cx37 antibodies (DF4079, Affinity BioSciences, Cincinnati, OH, USA) at 37 °C for 1 h, we performed a subsequent incubation for 1 h at 37 °C with the corresponding Alexa Fluor-linked secondary antibodies (Donkey-Antibody-Rabbit, Alx647, Jackon Immuno Research, West Grove, PA, USA). Negative controls were created by omitting primary antibodies from the procedure. After antibody incubation, sections were washed with PBS (pH 7.4, P5368, Sigma-Aldrich) and finally stained with a nuclear dye, DAPI (ab104139, Abcam, Cambridge, UK), for 10 min. The imaging and visualization of the fluorescent cells were performed using a Nikon C2 confocal microscope (Tokyo, Japan) with a 4× objective (confocal microscope zoom using 10× objectives).

### 2.3. PCR (Polymerase Chain Reaction)

The samples of fifty COCs, oocytes, and the corresponding cumulus cells were collected. Total RNA was extracted using the M5 Hiper Supermicroscale RNA Mini Kit (Mei5 Bioservices, Beijing, China) and RNA equivalent to the content of several samples was used for reverse transcription and cDNA amplification using the Prime Script TMRT reagent Kit (TaKaRa, Tokyo, Japan). For amplification of GJA4 cDNA, the upstream primer, 5′-CGACGAGCAGTCGGATTT-3′, and the downstream primer, 5′-AGATGACATGGCC CAGGTAG-3′, were used to amplify a 155-bp product. In accordance with the Premix TaqTM reagent instructions (RR901A, TakaRa), the cDNA was amplified in a PCR equipment (Applied Biosystems, Waltham, MA, USA), the reaction was pre-denatured at 95 °C for 30 s, followed by 39 cycles, each consisting of denaturation at 95 °C for 5 s, annealing at 60 °C for 30 s, extension at 72 °C for 15 s, and a final extension at 72 °C for 10 min.

### 2.4. ELISA

We randomly put eligible sheep COCs into the TCM-199 (Gibco) culture medium with or without Forskolin (100 μM) (F6886, Sigma) and IBMX (500 μM) (I5879, Sigma)—10 COCs in each group—and placed them into an in vitro culture in a 5% CO_2_ incubator at 39 °C for 0 min, 10 min, and 1 h. The cultured sheep COCs were collected into a 1.5 mL centrifuge tube, to which 150 μL of 0.1 M HCl was added for lysis, and the sample supernatant was collected. An aliquot of each sample was assayed for cAMP levels using a cAMP ELISA kit (581001, Cayman, UK) according to the manufacturer’s protocol. A multifunctional enzyme plate analyzer (CYT-1000, GE HealThCare, Chicago, IL, USA) was used to detect OD values at 412 nm, draw the standard curve, and calculate the cAMP level in samples.

### 2.5. Quantitative Real-Time PCR

Sheep COCs were placed in TCM-199 cultures containing Forskolin (100 μM) and IBMX (500 μM)—40 COCs per group—and cultured for 10 min at 39 °C in a 5% CO_2_ incubator under humidified air. The total RNA was extracted from each group and the cDNA was synthesized. qPCR was performed using 2 μL of amplified cDNA, 0.4 μM of specific primers (Table 1), and TB Green Premix Ex Taq™ II (RR820A, TakaRa) in a final reaction of 25 μL. The reactions were assayed in CFX96 Real-Time equipment (BIO-RAD, Hong Kong) and pre-denatured at 5 °C for 30 s, followed by 39 cycles of 95 °C denaturation for 5 s, annealing at 60 °C for 30 s, an extension at 72 °C for 15 s, and an extension at 72 °C for 10 min. We assessed the significance of the fold changes by comparing the averages 2^−ΔΔCt^ [12] values with a one-way ANOVA test.

### 2.6. Western Blotting

Sheep COCs were cultured in vitro for 10 min or 1 h after treatment with Forskolin (100 μM) plus IBMX (500 μM) or RP-cAMP (100 μM) in a hormone-free culture system. Forty COCs per group were put in an incubator containing 5% CO_2_ at 39 °C for cultivation.

Samples were washed and lysed with SDS (P1200, Solarbio) lysis buffer (containing protease inhibitors). Lysates were centrifuged and the protein concentration of supernatants was determined using Bio-Rad DC protein assay. Proteins were resolved on a 10% polyacrylamide gel and transferred to a nitrocellulose (EL32514, Pall, New York, NY, USA) membrane. NC-membranes were blocked with Odyssey Blocking fluid. For all proteins, 0.22 µm/well was loaded. For Cx37 immunoblotting, membranes were incubated with anti-Cx37 antibodies and anti-α-tubulin (Table 2) overnight at 4 °C. Membranes were then washed and further incubated with fluorophore-labeled anti-rabbit IgG antibody and anti-mouse IgG (Table 2) for 1 h at room temperature and later scanned using the Li-Cor Odyssey fluorescence scanner (SDS-PAGE electrophoresis system: 10% separating glue, 4% concentrated glue; 80 V constant pressure electrophoresis was used to concentrate the gel for 30 min, and 120 V constant pressure electrophoresis was used to separate the gel for 90 min. The membrane was transferred at a constant current of 380 mA for 90 min, ensuring low temperature during the entire transfer process).

### 2.7. Statistical Analysis

All experiments were performed at least three times from different batches of ovaries and with three replicates per batch. Data are presented as means ± SEM. Differences were analyzed by one-way analysis of variance (ANOVA) conducted using GraphPad Prism 5.0 (GraphPad Software). Significance was set at *p* < 0.05.

## 3. Results

### 3.1. Cx37 Expression in Sheep COC

**Figure 1:**
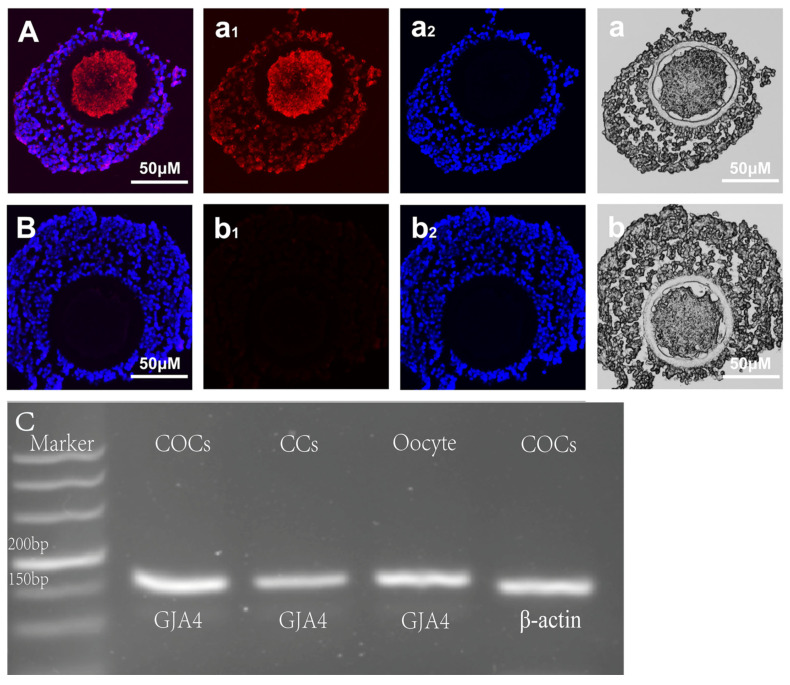
Expression of the Cx37 protein and GJA4 gene in sheep COC. Ovine COC section samples were stained for Cx37 using an Alx647 conjugated secondary antibody (Red) and nucleus using a DIPI (blue). (**A**) represents the combination of Cx37 and DIPI; (**a_1_**) and (**a_2_**) illustrate Cx37 and DIPI, respectively; (**a**) illustrates COCs morphology with normal light; (**B**), (**b_1_**), and (**b_2_**) are the negative comparison of A, (**a_1_**), and (**a_2_**) respectively; (**b**) shows COCs morphology with normal light; (**C**) illustrates the PCR electrophoresis results for the GJA4 gene. The size of the amplified product fragment of GJA4 gene in sheep COCs, CCs, and oocyte is 155 bp.

The new COCs paraffin section technology established in our laboratory was used to detect the expression of Cx37 protein in immature sheep COCs. The staining results show that the paraffin section retained the intact sheep COC structure, with both oocyte and cumulus cell layers clearly visible and the target protein located (Figure 1). The results show that the CX37 protein was located in both ovine oocytes and cumulus cells, as well as obvious cytoplasmic presence in ovine oocytes (Figure 1A). At the same time, the expression of the GJA4 gene in immature sheep oocytes and cumulus cells was detected by PCR. The results show that the amplified product bands were all target gene fragments, and the GJA4 gene was expressed in both sheep CCs and oocytes (Figure 1C).

### 3.2. The Effect of cAMP on the Expression of Cx37 in Sheep COC

**Figure 2:**
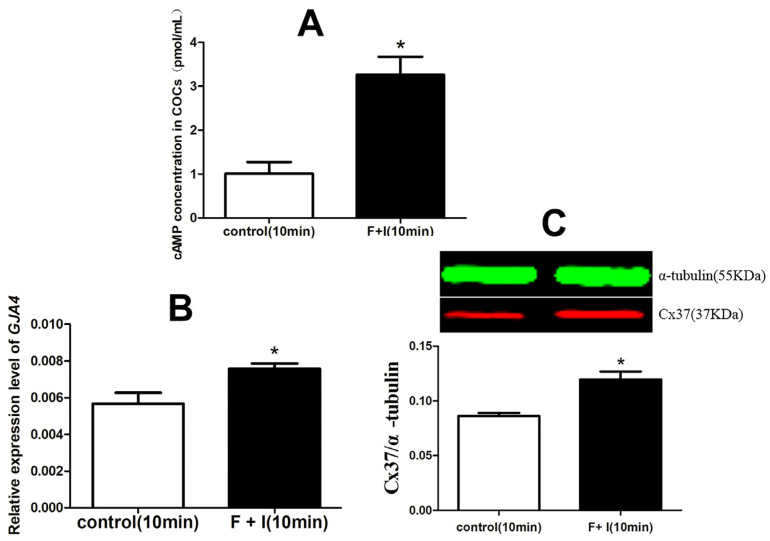
The effect of cAMP on the expression of Cx37 in sheep COC. (**A**) illustrated the effect of the cyclic adenosine monophosphate modulator on the level of cAMP in sheep COCs; (**B**) illustrates the effect of the cyclic adenosine monophosphate on the expression of the Cx37 gene in sheep COC; (**C**) illustrates the effect of the cyclic adenosine monophosphate on the expression of the Cx37 protein in sheep COC. F + I stands for Forskolin + IBMX, indicating the processing group. The experiment was repeated three times; * indicates a significant difference (*p* < 0.05).

In this experiment, a competitive ELISA method was chosen to detect cAMP concentrations in sheep COCs treated, or not, with Forskolin (100 μM) and IBMX (500 μM) for 10 min in a gonadotropin-free culture system. The results show that, compared to the control, Forskolin and IBMX treatment could increase the intercellular cAMP level significantly after culturing for 10 min in vitro (*p* < 0.05) (Figure 2A).

After immature sheep COCs were cultured with or without Forskolin plus IBMX for 10 min in vitro, the expression levels of both the GJA4 gene and the Cx37 protein were determined using qPCR and WB methods. The results show that Forskolin and IBMX were able to significantly promote the expressions of the GJA4 gene and the Cx37 protein when compared to the control (*p* < 0.05) (Figure 2B,C).

### 3.3. Effect of cAMP-PKA on Cx37 Protein

**Figure 3:**
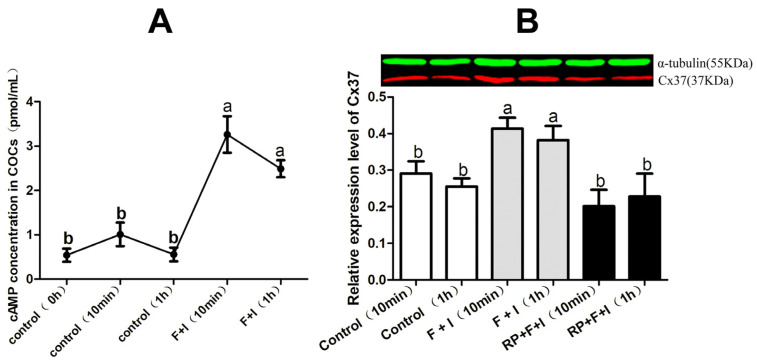
Effect of cAMP-PKA on Cx37 protein. (**A**) illustrates the effect of cyclic adenosine monophosphate modulator on the level of cAMP in sheep COCs; (**B**) illustrates the cyclic adenosine monophosphate regulating the expression of the Cx37 protein in sheep COCs. F + I and RP + F + I stand for Forskolin + IBMX and RP-cAMP + Forskolin + IBMX, respectively, representing the processing group. Forty COCs per group. Different letters indicate significant differences (*p* < 0.05).

In this experiment, ELISA was adopted to detect cAMP concentrations in sheep COCs treated, or not, with Forskolin (100 μM) and IBMX (500 μM) for 0 min, 10 min, and 60 min in a gonadotropin-free culture system. The results show that there was no significant difference in intercellular cAMP levels among the samples treated for different durations in vitro (*p* > 0.05). Compared to the controls, Forskolin and IBMX treatment significantly increased intercellular cAMP concentrations, whether the samples were incubated for 10 min or 60 min (*p* < 0.05) (Figure 3A).

Subsequently, western blotting was used to determine expression of the Cx37 protein in sheep COCs cultured in vitro for 10 min or 60 min with or without Forskolin plus IBMX or RP-cAMP. The results show that, compared to the corresponding control group, Forskolin and IBMX could significantly increase the level of Cx37 protein in sheep COCs when cultured for 10 min or 60 min in vitro (*p* < 0.05), a phenomenon which was neutralized after RP-cAMP addition (*p* < 0.05) (Figure 3B).

## 4. Discussion

Cx37 is expressed in oocytes and granulosa cells of a variety of mammals. Veitch [14] and Donfack et al. [15] found that Cx37 is present in the oocytes and granulosa cells of mice and goats, consistently with our results. In addition, other studies have shown that Cx37 may be located in oocyte cytoplasm [16]. In the preantral follicle stage of cattle, Cx37 is not only present in oocytes and GCs, but also shows diffuse staining in the ovarian cortex [17]. In addition, Grazull et al. [18] found that Cx37 is present in sheep ovarian endothelial cells, determined by cytoplasmic staining. In current sheep studies, Cx37 has been observed exclusively at the junctions between the CC and the oocyte. However, whether it solely originates from oocytes remains to be definitively determined [18,19]. We speculate that Cx37, mainly expressed in oocytes, is translated in the region near the nucleus and finally embedded in the oocyte membrane.

In order to verify the location and expression of the Cx37 protein in immature ovine COCs, we used PCR methods to detect the expression of the GJA4 gene in sheep oocytes, cumulus cells, and COCs. The results show that the GJA4 gene is expressed in sheep COCs, CCs and oocytes, a finding which is consistent with the abovementioned protein expression results. In addition, based on the rough judgment of the brightness of the target band, the expression level of the GJA4 gene in CCs seems to be lower than that of sheep oocytes, a finding which is consistent with other reports according to which Cx37 is mainly located in oocytes. Therefore, we speculate that Cx37 not only participates in the gap junction communication between the oocyte and its surrounding cells but is also involved in the formation of gap junction channels among cumulus cells. The GJC channel mediated by the Cx37 protein plays an indispensable role in mammalian oocyte maturation and follicular development. After the GJA4 gene is knocked out, the oocytes cannot restore meiosis, and the follicles cannot develop and reach the ovulation stage. Therefore, the gap junction channel formed by Cx37 is essential for oocytes to receive nutrition transmitted from cumulus cells and to synthesize RNA and proteins required for cytoplasmic maturation in the later stage. 

cAMP is a key regulator of mammalian oocyte meiotic arrest. It is well known that high levels of cAMP in oocytes are synergistically regulated by AC and PDE. Therefore, Forskolin and IBMX have been used in combination in many studies to regulate cAMP levels in COCs [20,21,22]. In sheep [23] and cattle [24] studies, the combined addition of Forskolin and IBMX at the same concentrations used in this study significantly increased cAMP levels in COC by more than 10 times. Margaret et al. [25] found that the combined addition of Forskolin and IBMX significantly increased the cAMP level in sheep COCs for 15 min to 60 min. Similarly to the above findings, in this study, the combined use of Forskolin and IBMX in a gonadotropin-free culture system for 10 min or 1 h significantly promoted the cAMP content in sheep COCs.

We subsequently explored the possible effects of cAMP modulators on the expression of Cx37 mRNA and protein in sheep COCs. The results indicate that, after culturing in vitro for 10 min, Forskolin and IBMX treatment significantly increased the expression levels of CX37 mRNA and protein in sheep COCs. At present, there are few reports on the relationship between cAMP and Cx37 expression during follicular development and oocyte maturation in sheep and other species. Li Haijun et al. [26] found that the use of the same concentration of cAMP modulator as in this experiment can not only significantly prolong the GJC opening time, but also enhance the GJC function between bovine COC. In another related human study, the use of Cilostamide and Forskolin induced cAMP accumulation, alleviated GJC loss, caused a delay in GVBD, and improved oocyte quality [13]. Therefore, it is speculated that cAMP-mediated GJC is involved in Cx37 expression in ovine oocytes during maturation in vitro.

However, Sela et al. [27] incubated rat follicles with CBX and found that almost all follicle-enclosed oocytes initiated meiotic recovery after 5 h of culture. Our results show that the enhancement of GJC function may be at least partly associated with the positive regulatory effect of cAMP on Cx37 expression. It is well known that cAMP plays an important role in maintaining oocyte meiotic arrest. Therefore, during oocyte maturation, cAMP enhances oocyte–cumulus cell GJC through upregulation of Cx37 expression in ovine COCs; subsequently, intraoocyte cAMP accumulation through continuously open junction channels become one of the key prerequisites for maintaining meiotic arrest. 

In order to further explore the possible mechanism of cAMP with respect to the promotion of Cx37 expression in sheep COCs, RP-cAMP was used, competitively blocking the activating effect of cAMP on PKA activity and significantly downregulating the gene and protein expressions of Cx37 in ovine COCs. Vivek [28] found that the specific inhibitor of PKA, H89, could significantly reduce the expression of the Cx43 protein mediated by CBN. Keiichiro [29] reported that FSH could induce the phosphorylation of the Cx43 protein in rat granulosa cells—a process which could be counteracted after PKA activity was inhibited with H89—and positively regulate the formation and activity of Cx43-mediated channel at the PKA-specific phosphorylation site. Another study reported that short-term (10 min) exposure of rat follicles to an LH stimulus was able to phosphorylate Cx43, and the phosphorylation process was regulated by PKA and PKC signaling pathways [30]. Most of these studies focused on the regulation of PKA kinase with respect to the expression or function of Cx43, but the correlation between PKA kinase and Cx37 expression was rarely reported. Our results suggest that the positive effect of the regulation by cAMP on CX37 protein and gene expression in sheep COCs might be related to the PKA signaling pathway. This finding is similar to the abovementioned Cx43-related research results. As the two main types of gap junction proteins in COCs, Cx37 and Cx43 together form the gap junction channel between the cumulus cell and the oocyte, and jointly mediate the process of information and material exchange among cells.

## 5. Conclusions

Cx37 protein and mRNA were detected in both immature sheep oocytes and cumulus cells. Forskolin and IBMX treatment significantly increased Cx37 expression in a PKA-dependent manner in sheep COC cultured in vitro.

## Figures and Tables

**Table 1:** GJA4 and β-actin primer sequence.

Primer Name	Primer Sequences (5′–3′)	Fragment Length
GJA4	F-CGACGAGCAGTCGGATTT R-AGATGACATGGCCCAGGTAG	155 bp
β-actin	F-CCATCGGCAATGAGCGGT R-CGTGTTGGCGTAGAGGTC	146 bp

**Table 2:** Antibody information.

Primary Antibodies	Firm	Host	Dilution Ratio	Protein Molecular Weight	Secondary Antibodies
Cx37	Affinity	Rabbit	1:500	37 KDa	IRDye^®^680CW (Donkey anti-Rabbit 1:10,000)
α-tubulin	Abcam	Mouse	1:20,000	55 KDa	IRDye^®^800CW (Donkey anti-Mouse 1:10,000)

## Data Availability

The data that support the findings of this study are available upon request from the corresponding author.
